# *BRAF*/*NRAS* wild-type melanoma, NF1 status and sensitivity to trametinib

**DOI:** 10.1111/pcmr.12316

**Published:** 2014-10-13

**Authors:** Marco Ranzani, Constantine Alifrangis, Daniele Perna, Ken Dutton-Regester, Antonia Pritchard, Kim Wong, Mamunur Rashid, Carla Daniela Robles-Espinoza, Nicholas K Hayward, Ultan McDermott, Mathew Garnett, David J Adams

**Affiliations:** 1 Experimental Cancer Genetics, The Wellcome Trust Sanger InstituteHinxton, Cambridge, UK; 2 Cancer Genome Project, The Wellcome Trust Sanger InstituteHinxton, UK; 3 Oncogenomics Laboratory, QIMR Berghofer Medical Research InstituteBrisbane, Qld, Australia

Dear Editor,

Despite recent advances in the management of metastatic melanoma using targeted therapies, options for patients with tumours that are *BRAF* and *NRAS* wild type remain limited (Dummer et al., 2012). *BRAF*/*NRAS* wild-type melanoma accounts for 13–26% of all melanoma cases (Hodis et al., 2012; Mar et al., 2013) and is generally characterized by a high C > T mutation burden, loss of function mutations and deletions of *NF1*, and activating mutations of *KIT*. Amplification of *KIT, CCND1* and *TERT* is also observed in this disease (Hodis et al., 2012; Mar et al., 2013). Dacarbazine chemotherapy is the standard of care for patients with this molecular class of melanoma, but response rates in advanced disease are disappointing (Dummer et al., 2012; Tsao et al., 2004). More recently, immunotherapy has been deployed for this disease, eliciting marked responses in a subset of patients, but in most only a modest improvement in survival over chemotherapy has been observed (Robert et al., 2011) (see also J Clin Oncol 31, 2013; suppl; abstr 9025). In the subset of patients with *BRAF*/*NRAS* wild-type melanoma carrying *KIT* mutations, KIT inhibitors have shown some efficacy, particularly in patients with exon 11 or 13 mutations (Goldinger et al., 2013). This therapeutic modality is, however, only applicable to the 10–22% of patients with *KIT* mutant *BRAF*/*NRAS* wild-type disease (Hodis et al., 2012; Mar et al., 2013). Collectively, the survival of patients with metastatic *BRAF*/*NRAS* wild-type melanoma remains dismal.

Trametinib (a competitive MEK1/2 inhibitor) alone or in combination with BRAF inhibitor treatment has significantly improved the survival of patients with *BRAF* mutant melanoma (Flaherty et al., 2012). Additionally, some patients with *NRAS* mutant disease have been shown to respond to MEK inhibitor-based therapy (Ascierto et al., 2013). Although partial responses have been described in patients with *BRAF*/*NRAS* wild-type melanoma in a Phase 1 clinical trial of trametinib, the validity of this therapy has not been fully explored in this subclass of disease (Falchook et al., 2012). Recently, Nissan et al. (2014) showed that trametinib efficiently inhibited cell growth and ERK signalling in *BRAF*/*NRAS* wild-type melanoma cell lines that had lost NF1*,* a negative regulator of RAS signalling. As 56–76% of *BRAF*/*NRAS* wild-type melanomas do not carry loss of function mutations of *NF1* (Hodis et al., 2012; Mar et al., 2013), we investigated the sensitivity to trametinib of cell lines retaining NF1 expression.

We assembled a collection of 25 patient-derived melanoma cell lines and determined their mutational status for a panel of 19 melanoma cancer genes (Table S1). Our collection comprised 9 cell lines carrying activating mutations of *BRAF* and 16 *BRAF/NRAS* wild-type cell lines (Table S1). The sensitivity of each cell line to trametinib was assessed using Syto60, a nucleic acid-based assay, after 6 days of exposure to 9 different escalating doses of trametinib (range 0.08–10 nM). This assay provides a robust estimate of cell viability (Garnett et al., 2012), and is consistent with live cell assays (see Figure S1 and Data S1). All *BRAF*/*NRAS* wild-type melanoma lines displayed a IC50 for trametinib in the nanomolar range, which was comparable to the IC50 for the *BRAF*-mutated cell lines that were tested in parallel (mean IC50 ± standard error mean = 2.54 nM ± 0.85 and 2.46 nM ± 1.05 for *BRAF*/*NRAS* wild-type and *BRAF*-mutated melanomas, respectively; P = 0.96 by two tailed unpaired *t*-test; Figure1A and Table1). Compared to the IC50 of a panel of 316 cancer cell lines screened for trametinib sensitivity, *BRAF*/*NRAS* wild-type melanoma lines are scored as highly sensitive (Figure S2 and Table S2) suggesting that utilisation of the MAPK pathway is an intrinsic feature of these melanomas. These results confirm and extend the validity of a recent study showing that *BRAF*/*NRAS* wild-type melanoma cell lines are sensitive to trametinib and suggest that they can be as sensitive to MEK inhibition as melanomas with *BRAF* mutations (Stones et al., 2013). To further stratify the *BRAF/NRAS* wild-type melanomas in our cell line collection, we assessed NF1 status by Western blotting (see Data S1) and sequencing (Table S1). Nine of the 16 *BRAF/NRAS* wild-type cell lines analysed by Western blotting displayed undetectable NF1 protein levels while 7 expressed NF1 protein (Figure1B, Table1 and Table S1). Remarkably, the 7 *BRAF*/*NRAS* wild-type melanoma cell lines that expressed NF1 protein showed a similar sensitivity to trametinib as cell lines in which NF1 protein was undetectable (IC50 1.81 nM ± 1.20 and 3.10 nM ± 1.22 for NF1-positive and NF1-negative melanomas, respectively; P = 0.47 by two tailed unpaired *t*-test; Figure1C and Table1). To confirm the effectiveness of MEK inhibition by trametinib in cell lines of different NF1 expression status, we measured the levels of phospho-ERK, a downstream effector of the MAPK pathway. Treatment with escalating doses of trametinib (0.01–10 nM) revealed reduced levels of phospho-ERK at 1 and 10 nM trametinib in all the cell lines tested (Figure1D and Figure S3). We then measured the expression levels of downstream transcriptional targets of ERK: *ETV5* and *PHLDA1* (see Data S1). Trametinib induced a significant decrease of *ETV5* and *PHLDA1* levels in 3/4 and 4/4 cell lines, respectively (Figure1E). Overall, these results show that trametinib induces a functional downregulation of the ERK pathway (Figure 1D–E) in *BRAF/NRAS* wild-type melanoma cell lines, and that lines that express NF1 protein can also be defined as sensitive to MEK inhibition.

**Table 1:** Sensitivity of the 25 melanoma cell lines to trametinib and their mutation status

Cell line ID	NRAS	BRAF	NF1 PROTEIN	TRAMETINIB ICI50 (nM)
C058	WT	L597P	NA	0.15
M14	WT	V600E	NA	0.6
C32	WT	V600E	NA	0.7
HT144	WT	V600E	NA	1
MR1010B	WT	V600E	NA	2.5
A101D	WT	V600E	NA	3
IGR1	WT	V600E	NA	4
ISTMEL1	WT	V600E	NA	>10
C089	WT	V600E	POSITIVE	0.2
D35	WT	WT	POSITIVE	0.4
A04	WT	WT	POSITIVE	0.4
C052	WT	WT	POSITIVE	0.5
C037	WT	Translocation	POSITIVE	0.5
D10	WT	WT	POSITIVE	0.6
D38	WT	WT	POSITIVE	1.3
CHL-1	WT	WT	POSITIVE	9
Colo-792	WT	WT	NEGATIVE	0.4
C008	WT	WT	NEGATIVE	0.6
C067	WT	WT	NEGATIVE	0.7
C025	WT	WT	NEGATIVE	0.9
MeWo	WT	WT	NEGATIVE	1
C086	WT	WT	NEGATIVE	1.3
C077	WT	WT	NEGATIVE	5
C021	WT	WT	NEGATIVE	8
D24	WT	WT	NEGATIVE	10

Each cell lines was treated with nine different concentrations of trametinib (range 0.08–10 nM) for 6 days, fixed and stained with Syto60. The relative viability was calculated versus the vehicle treated control. The IC50 was calculated from the dose response curve. Experiments were performed in triplicate. The mutation status was assessed by Sequenom MassArray platform. NF1 protein expression was evaluated by Western blotting, as described in Figure1. NA = not analysed. C037 cell line carries a *BRAF* translocation (see Data S1). See also Table S1.

**Figure 1:**
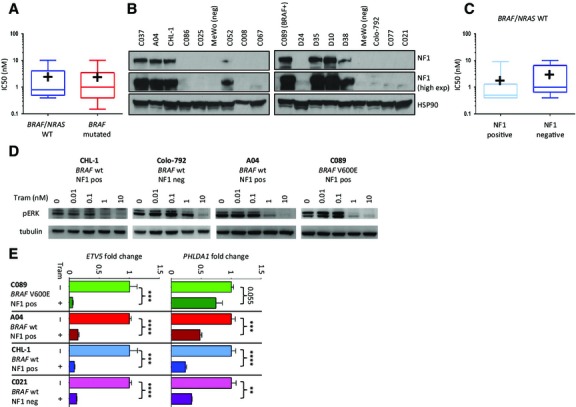
*BRAF*/*NRAS* wild-type melanoma cell lines, NF1 expression and sensitivity to trametinib. (A) The IC50 values for trametinib in *BRAF/NRAS* wild-type melanoma cell lines are comparable to those that are *BRAF* mutant. The box extends from the 25th to 75th percentiles, the whiskers from the minimum to the maximum value. The horizontal line represents the median, the ‘+’ shows the mean. (B) Western blot analysis of NF1 protein (expected 250 kD band in the upper panel). The HSP90 protein level in the lower panel was used as loading control. The cell line ID is shown above the blots. (C) The sensitivity to trametinib of NF1 negative melanoma cell lines is comparable to those that express NF1. Data are graphed as in A. (D) Treatment with trametinib at escalating doses for 6 h induces downregulation of p-ERK in *BRAF*/*NRAS* wild-type cell lines (upper panel). C089 represents a *BRAF* V600E mutant control. Tubulin was used as loading control. Cell line ID and mutation status are shown above the blots. (E) Treatment with trametinib 10 nM for 24 h induced the downregulation of *ETV5* and *PHLDA1*, ERK target genes, in four representative melanoma cell lines. Gene expression was detected by real-time PCR, using *β*-actin as a housekeeping control. Fold expression is shown relative to vehicle-treated control cells. *P* values by unpaired *t*-test, ** = P < 0.01, *** = P < 0.001, **** = P < 0.0001. See also Data S1.

In summary, we show that *BRAF*/*NRAS* wild-type melanomas are highly sensitive to the MEK inhibitor trametinib, and that loss of NF1 protein expression alone does not stratify sensitive cell lines. Overall, our findings mandate further investigation of the efficacy of trametinib in *BRAF*/*NRAS* wild-type melanoma. Given the limited therapeutic options for *BRAF*/*NRAS* wild-type melanoma, trametinib may represent a useful therapeutic tool for patients with this subclass of the disease.

## Supporting Information

Figure S1. The Syto60 nucleic acid assay provides a measurement of cell viability that is comparable to two independent live cell assays.

Figure S2. Sensitivity to trametinib of a panel of 316 cancer cell lines.

Figure S3. Trametinib induced p-ERK downregulation in melanoma cell lines independent of their *BRAF/NRAS* and *NF1* status.

Table S1. Mutation status of the 25 melanoma cell lines.

Table S2. Sensitivity to trametinib of a panel of 316 cancer cell lines.
