# Supplementary material for: BRAF/NRAS wild-type melanoma, NF1 status and sensitivity to trametinib
Source: Pigment Cell Melanoma Res. 2014 Oct 13;28(1):117–9. doi: 10.1111/pcmr.12316 (PMC4296225; doi:10.1111/pcmr.12316)
Supplement: Supplementary file 1 — Figure S1. The Syto60 nucleic acid assay provides a measurement of cell viability that is comparable to two independent live cell assays. [file pcmr0028-0117-sd1.pdf]

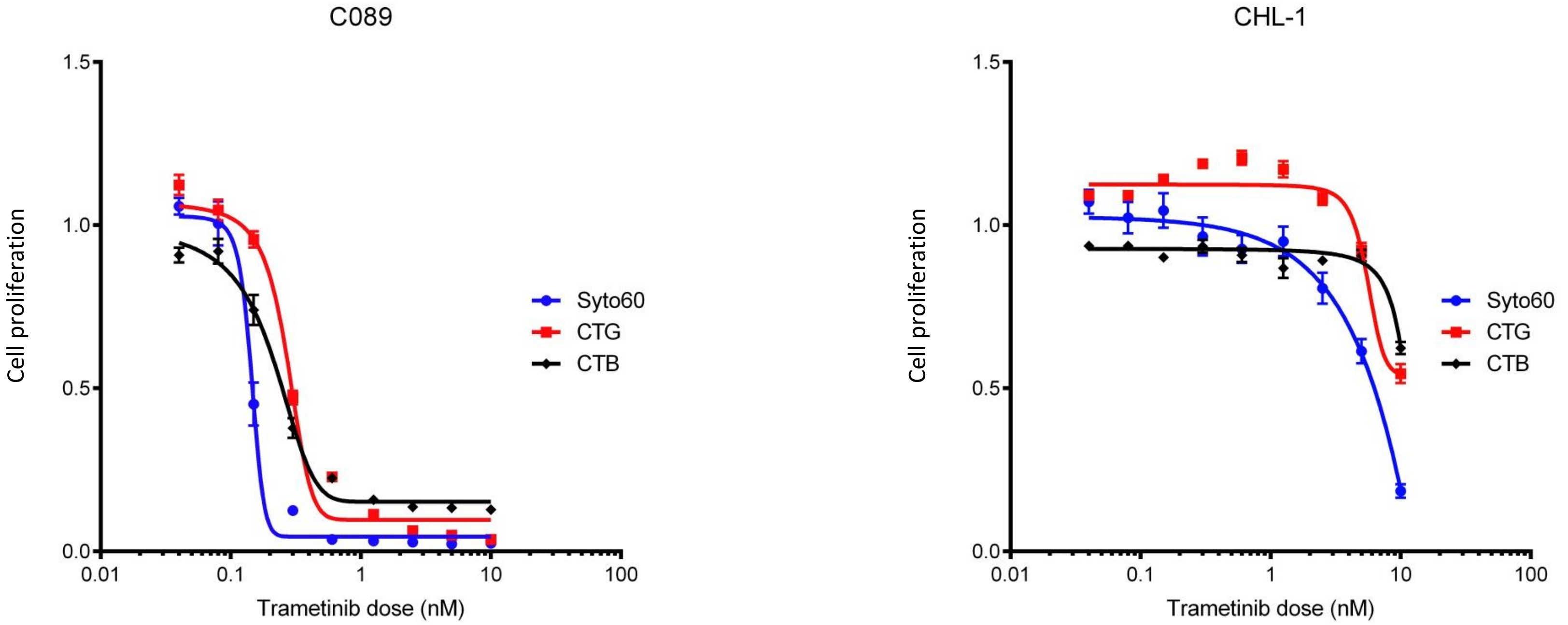

Supplementary Figure 1. The Syto60 nucleic acid assay provides a measurement of cell viability that is comparable to two independent live cell assays. C089 and CHL-1 cells were seeded in 96 well plates at predetermined optimal densities. The cells were treated with 9 different concentration of trametinib (0.04-10nM) for 6 days. The 3 assays were performed on 3 sister 96 well plates treated simultaneously with the same drug dilutions. Cell proliferation (Y axis) is calibrated on the vehicle treated control. Trametinib concentration (X axis) is expressed in nM concentration in logarithmic scale. Dots represent the mean of 3 experimental replicates, whiskers indicate the standard error of the mean. Solid lines represent the non-linear curve fitting by Graphpad Prism. CTG = Cell Titer Glo assay; CTB = Cell Titer Blue assay (see Supplementary Methods).
