# Supplementary material for: BRAF/NRAS wild-type melanoma, NF1 status and sensitivity to trametinib
Source: Pigment Cell Melanoma Res. 2014 Oct 13;28(1):117–9. doi: 10.1111/pcmr.12316 (PMC4296225; doi:10.1111/pcmr.12316)
Supplement: Supplementary file 2 — Figure S2. Sensitivity to trametinib of a panel of 316 cancer cell lines. [file pcmr0028-0117-sd2.pdf]

Number of cell lines screened	316
Maximum IC50 (µM)	178
Geometric mean	0.405
Minimum IC50 (µM)	0.000025
Min screening concentration (µM)	0.00391
Max screening concentration (µM)	1

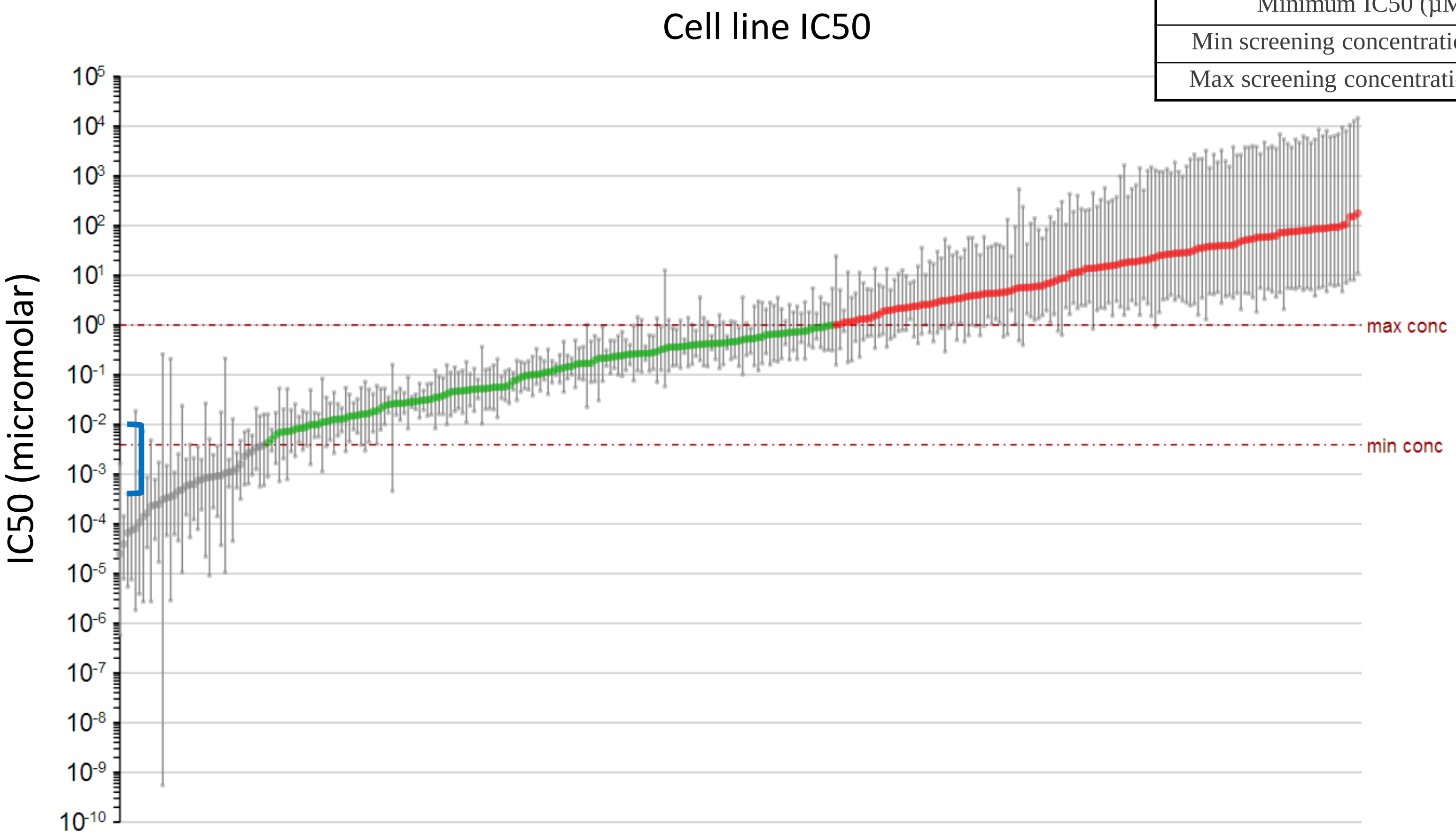

Supplementary Figure 2. Sensitivity to trametinib of a panel of 316 cancer cell lines. The cell lines are ranked along the x axis according to the IC50, which is shown on the Y axis in logarithmic scale. Whiskers represent the confidence interval. Green colour highlights cell lines with an IC50 within the screening concentration range, grey below the minimum screened concentration, red above the maximum screened concentration. The blue parenthesis highlights the IC50 range for the *BRAF*/*NRAS* wild type melanomas. Each cell line was treated with 9 different concentrations of trametinib (2 fold dilution each) for 3 days. Cell proliferation was measured by Syto60 and IC50 was estimated from the curve fitted data points according to the algorithm described in Garnett et al. Nature 2012. See also Table S2 for additional details on the cell lines.
