# Supplementary material for: BRAF/NRAS wild-type melanoma, NF1 status and sensitivity to trametinib
Source: Pigment Cell Melanoma Res. 2014 Oct 13;28(1):117–9. doi: 10.1111/pcmr.12316 (PMC4296225; doi:10.1111/pcmr.12316)
Supplement: Supplementary file 3 — Figure S3. Trametinib induced p-ERK downregulation in melanoma cell lines independent of their BRAF/NRAS and NF1 status. [file pcmr0028-0117-sd3.pdf]

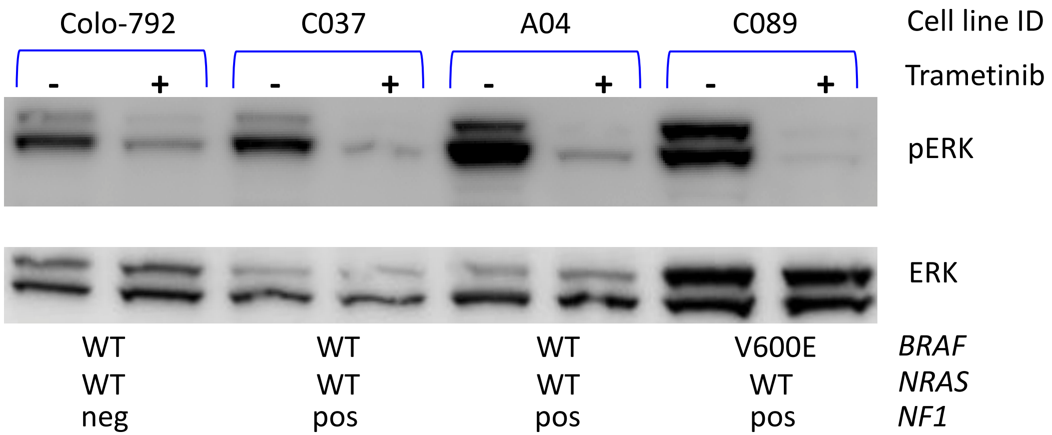

Supplementary Figure 3. Trametinib induced p-ERK downregulation in melanoma cell lines independent of their *BRAF/NRAS* and *NF1* status. Treatment with trametinib 5nM for 6h induces downregulated p-ERK in *BRAF*/*NRAS* wild type NF1 positive cell lines, and in controls.
