# Supplementary material for: BRAF/NRAS wild-type melanoma, NF1 status and sensitivity to trametinib
Source: Pigment Cell Melanoma Res. 2014 Oct 13;28(1):117–9. doi: 10.1111/pcmr.12316 (PMC4296225; doi:10.1111/pcmr.12316)
Supplement: Supplementary file 6 — Data S1. Methods. [file pcmr0028-0117-sd6.docx]

**Supplementary Methods**

**Cell proliferation assays**

Following seeding density optimisation for each cell line, the sensitivity to trametinib was measured using the Syto60 (Life Technologies) nucleic acid stain assay according to the protocol detailed in *Garnett MJ*, *Nature 2012*. This assay estimate the number of cells at the endpoint of the experiment by SYTO 60 Red Fluorescent Nucleic Acid Stain (Life Technologies; http://www.lifetechnologies.com/order/catalog/product/S11342), which is a cell permeant dye with bright red fluorescence (excitation 652nm, emission 678nm) upon binding to nucleic acid. Briefly, 24h after seeding in 96 well plates 9 different concentrations of trametinib (two-fold dilutions in the range 0.08-10nM plus DMSO vehicle only) was added to cells in culture. Six days after drug treatment the cells were fixed with formaldehyde 4% for 20 minutes, washed twice in dH_2_O, stained with SYTO 60 at 1:5000 for 1 hour, and washed twice in dH_2_O. Each experiment was performed using biological triplicates. Quantification of fluorescent signal intensity was then performed using a fluorescent plate reader. Cell proliferation was calculated normalizing for the vehicle-treated cells. IC50 was calculated from the proliferation curves.

In order to confirm the robustness of the assay, we performed a comparison between the Syto60 assay, Cell Titer Glo assay (based on the quantitation of cellular ATP; Promega, https://www.promega.co.uk/products/cell-health-and-metabolism/cell-viability-assays/celltiter_glo-luminescent-cell-viability-assay), and the Cell Titer Blue assay (estimating cell metabolic activity as a measure of resazurin reduction; Promega; https://www.promega.co.uk/products/cell-health-and-metabolism/cell-viability-assays/celltiter_blue-cell-viability-assay) on 2 cell lines that cover the extremes of sensitivity to trametinib from our collection. Cells were seeded as described above in 3 replicate 96 well plates and after 6 days of treatment with trametinib, each plate was stained with Syto60 as described above, or with Cell Titer Glo or Cell Titer Blue assays according to the manufacturer’s instructions. Quantification of fluorescent signal intensity was then performed using a fluorescent plate reader. Cell proliferation was calculated normalizing for the vehicle-treated cells.

**Western blotting**

Cell lysis was performed in culture dishes with NP40 Cell Lysis Buffer (Life Technologies) supplemented with a Protease/Phosphatase Inhibitor Cocktail (Cell Signalling). Lysates were cleared by centrifugation at 13000 rpm for 10 minutes at 4°C. For NF1 detection (Figure 1B), 20µg of protein was loaded on a Polyacrylamide gel and transferred to PVDA membrane prior to immunoblotting with an anti-NF1 antibody (Bethyl Laboratories A300-140A, used 1:1000) or HSP90 as protein loading control (anti-HSP90 1:500 Cell Signaling #4875). Signal was detected using a HRP-conjugated secondary antibody and autoradiography films.

For p-ERK detection (Figure 1D), 20ug of protein was loaded on a Polyacrylamide gel and transferred to PVDA membrane prior to immunoblotting with an antibody against p-ERK (1:1000, Cell Signalling 9101), or tubulin as protein loading control (1:2500, Sigma T8238). In Figure S3 total ERK was detected with a primary antibody raised against ERK (1:1000, Cell Signalling 9102). Signal was detected using a HRP-conjugated secondary antibody on a Genesnap digital scanner.

**Gene expression**

Total RNA was collected from cell lines treated for 24h with 10nM trametinib. RNA was isolated using the RNeasy Mini Kit (Qiagen). cDNA preparation was performed using Mo-MLV reverse transcriptase and random hexamers primers (Invitrogen, Superscript III First-Strand Synthesis System for RT-PCR). Real time PCR was performed with SYBR Green PCR Master Mix (Life Technologies) and the following oligos:

| **Gene** | **Oligo ID** | **sequence 5' 3'** |
| --- | --- | --- |
| *ETV5* | ETV5_Fw | catcctacatgagagggggtta |
| *ETV5* | ETV5_Rv | caaagtataatcggggatctttttc |
| *PHLDA1* | PHLDA1_Rv | CTTCTCCGCAGCACCTCCAA |
| *PHLDA1* | PHLDA1_Fw | TTCCTCAAGTCCTCAAAACCTTGT |
| *ACTB* | ACTBf | CATGTACGTTGCTATCCAGGC |
| *ACTB* | ACTBr | CTCCTTAATGTCACGCACGAT |

Amplification reactions were performed on an ABI7900 Real-time PCR thermal cycler using a standard amplification protocol followed by the generation of a dissociation curve to check the specificity of the amplification. Non-retrotranscribed control reactions confirmed that amplification was transcript specific and not due to genomic DNA contamination. The relative expression level of each gene was calculated using the ^ΔΔ^Ct method (*Pfaffl, NAR 2001*), normalized to β-actin expression (housekeeping control gene), and represented as fold change relative to average of the vehicle treated biological triplicate (calibrator). Each experimental point was performed in biological triplicate; each gene expression was performed in technical triplicate.

**Cell line mutation status**

Cell lines mutations are summarized in Table S1. The mutation status for *NRAS, BRAF, AKT1, AKT3, PIK3CA, MET, PDGFRA, RAC1, NEK10, ERBB4, MEK, BCL2L12, PPP6C, TRRAP* was determined for the mutations indicated using the Sequenom MassArray platform, while the mutation status for *MAP3K9, MC1R, PTEN, p14ARF, TP53* was determined by capillary sequencing. *NF1* mutations were identified following *NF1* capture sequencing. Non-synonymous mutations and essential splicing site mutations are reported; mutation coordinates are indicated for the *NF1* transcript NM_001042492 for exonic mutations, and as genomic coordinates for the splicing mutations. Bold font highlights mutations also found in COSMIC. Different mutations are separated by semi colon. The mutation status of C32, IGR1, A101D, HT144, ISTMEL1, CHL-1, Colo-792, MeWo cell lines was determined by the Cancer Cell Line Project whose data is available via COSMIC; full mutation data for these cell lines is available at https://cansar.icr.ac.uk/cansar/cell-lines/#main_tab_holder:tab_search_main_protein:tab_search_1.

For 13 of the 16 *BRAF*/*NRAS* wt melanoma cell lines we have RNA sequencing data (data not shown). This analysis revealed that the C037 cell line carries a *BRAF* translocation.
